# Microencapsulation of *Ruellia tuberosa* L. Aqueous Root Extracts Using Chitosan-Sodium Tripolyphosphate and Their In Vitro Biological Activities

**DOI:** 10.1155/2022/9522463

**Published:** 2022-06-01

**Authors:** Anna Safitri, Anna Roosdiana, Nia Kurnianingsih, Fatchiyah Fatchiyah, Eldina Mayasari, Rina Rachmawati

**Affiliations:** ^1^Department of Chemistry, Faculty of Mathematic and Natural Sciences, Brawijaya University, Malang 65145, Jl. Veteran, Indonesia; ^2^Research Center for Smart Molecules of Natural Genetic Resources (SMONAGENES), Brawijaya University, Malang 65145, Jl. Veteran, Indonesia; ^3^Department of Physiology, Faculty of Medicine, Brawijaya University, Malang 65145, Jl. Veteran, Indonesia; ^4^Department of Biology, Faculty of Mathematic and Natural Sciences, Brawijaya University, Malang 65145, Jl. Veteran, Indonesia

## Abstract

The current study aims to perform microencapsulation of *R*. *tuberosa* L. extracts using chitosan crosslinked to sodium tripolyphosphate (NaTPP) as wall materials by spray drying and to analyze their in vitro biological activities. The influence of manufacturing conditions, like pH, chitosan concentration, and stirrer time, was assessed. Results showed that microcapsules prepared in pH 4 with a concentration of 0.1% (w/v) chitosan, and 90 min stirring time had 51.80% encapsulation efficiency and high in vitro biological activity. These were shown by high in vitro alpha amylase inhibition and antioxidant activity with IC_50_ values of 50.65 *μ*g/mL and 123.97 *μ*g/mL, respectively. Releases of the bioactive compounds in microcapsules of *R*. *tuberosa* L. were carried out on phosphate buffer medium pH 2.2 and pH 7.4 with times release of 30, 60, 90, and 120 min. The bioactive compounds were released in pH 2.2 in 120 min at 2.48%. At pH 7.4, the active ingredients were more easily released, by 79.90% in 120 min. The microcapsules' morphology showed a rough surface with spherical forms and the average sizes were 53.41 *μ*m. This study supports the essential role of microencapsulation in improving plant extracts with reserved biological activities.

## 1. Introduction

Exploration of bioactive compounds from natural materials can contribute to knowledge in the health sector, both in preventing and treating of diseases [1, 2]. Indonesia has a very abundant diversity of plants that can be utilized to supply raw ingredients for traditional medicine [3, 4]. One of the plants that can be used as a resource of bioactive compounds is from the Acanthaceae family. Genus *Ruellia* is one of the members of the Acanthaceae family. *Ruellia tuberosa* L. is broadly spread in Asian countries (i.e., Indonesia) [5]. This plant contains a large number of phytochemicals, including flavonoids, terpenoids, phenols, and other compounds containing antidiabetic potential [6, 7]. This plant has also been applied in several in vivo studies as antidiabetic agent [8, 9]. Therefore, producing an ingredient containing *R*. *tuberosa* L. extract is necessary. However, phytochemicals such as flavonoids, terpenoids, and phenols are very sensitive to environmental conditions, such as temperature or oxygen; hence, microencapsulation was supposed to protect these components [10, 11].

The spray-drying process has been one of the popular techniques for decades to encapsulate natural products because of its low operational cost and available equipment [12, 13]. It has been used to produce powder from a liquid form by using carrier agent materials. Chitosan, a partially deacetylated chitin derivative composed of *N*-acetylglucosamine, has emerged as a significant biomaterial and pharmaceutical excipient for drug delivery because of its biocompatibility, biodegradability, low immunogenicity, and low cost [14]. In addition to its low toxicity, its hemostatic and bacteriostatic properties also contribute to its pharmaceutical applications [15].

In the preparation of microcapsules, several factors including stirring time, stirring speed, temperature, pH, and concentration of the core material contribute to the microcapsule's properties [16, 17]. The coating concentration greatly affects the particle size and the efficiency of the microencapsulation [18, 19]. Furthermore, chitosan as a coating material is generally pH dependent, since chitosan dissolves in weak organic acid solvents in the pH ranges of 4–6.5 [20]. The present study focused on the preparation of spray-drying microencapsulation and evaluate the biological activities of microencapsulated *R*. *tuberosa* L. extracts.

Since extracts of *R*. *tuberosa* L. have antidiabetic activity, the biological activities can be determined through antioxidant activity assay and their inhibition to alpha amylase. The inhibition of the enzyme involved in the hydrolyzing carbohydrates, such as alpha amylase, is important for reducing hyperglycemia [21]. Alpha amylase (E.C.3.2.1.1) is a potential protein tending to be applied as inhibitor for antidiabetic treatment [22]. In human beings, alpha amylase is a prominent enzyme which hydrolyses the alpha bonds of large, alpha-linked polysaccharides, such as starch and glycogen, yielding glucose and maltose [23, 24]. The inhibition of alpha amylase has minimized the absorption of glucose into the blood by delaying the digestion of carbohydrates [24]. In addition, based on previous research, aqueous *R*. *tuberosa* L. extract contains flavonoids, phenolics, and tannins that can function as antioxidants [25]. Therefore, it is interesting to investigate whether the microencapsulated aqueous extracts of *R*. *tuberosa* L. still retained their biological activities.

## 2. Materials and Methods

### 2.1. Chemical and Reagents

The research materials used were purchased from Merck: soluble starch (from potato, ACS grade), 3,5-dinitrosalicylic acid (DNS) reagent (≥98%, HPLC grade), acarbose (≥95%), glacial acetic acid (pharmaceutical primary standard), alpha amylase from *Aspergillus oryzae* (≥150 units/mg protein), chitosan (low molecular weight, 50,000–190,000 Da), sodium tripolyphosphate (NaTPP, technical grade, 85%), D-(+) glucose (analytical standard), quercetin (≥95% HPLC, solid), ascorbic acid (pharmaceutical secondary standard), sodium hydroxide (≥98%, pellets, anhydrous), potassium sodium tartrate tetrahydrate (≥99%), sodium sulfite (≥98%), and sodium acetate (anhydrous, ≥99%). The *R*. *tuberosa* L. root powder was obtained from UPT Materia Medica Batu, East Java, Indonesia, enclosed with determination letter of species.

### 2.2. Extract Preparation

*Ruellia tuberosa* L. root powder was extracted based on previous study [25], using maceration technique, with preheated distilled water, in the volume of 4x dried weight, for 2 × 24 h. A 100 grams of *R*. *tuberosa* L root powder was used, with 400 mL of distilled water as solvent. The resulted extracts were collected through filtration. To obtain the concentrated extracts, a rotary evaporator vacuum was used with a slow speed at 110 rpm, at 65°C, for about 6 h. The concentrated extracts were kept at 4°C for subsequent analysis. The characterization of the extracts has been conducted in our earlier published work [25].

### 2.3. Microencapsulation Procedures

The *R*. *tuberosa* L. extract (0.5 g) was dissolved with 17.5 mL of distilled water. Then, 50 mL of 1% chitosan solution (w/v) in 2% acetic acid (v/v, pH variations of 3, 4, 5, and 6) was added slowly and stirred with a magnetic stirrer for 60 min at a speed of 500 rpm. After that, 175 mL of NaTPP 0.3% (w/v) was added slowly and stirred again with a magnetic stirrer for 60 min. The colloid microcapsules in chitosan and NaTPP were then dried using a mini spray dryer Buchi B-290 with a standard 0.5 mm nozzle. The inlet temperature was 105°C, and the outlet temperature was 85°C, an air pressure of 1 bar, liquid flow rate of 1.5 mL/min, and a spray air flow of 400 LN/h. The process was repeated under the influence of different concentrations of chitosan solution at 0.05%, 0.1%, 2%, and 3% (w/v). The initial pH that contributed to the optimum encapsulation efficiency was used, whereas other conditions were the same. Finally, the effect of stirring time was determined by repeating the process using different stirring times at 60, 90, 120, and 150 min. The concentration of NaTPP were not varied throughout preparation of the microcapsules, and 0.3% (w/v) was chosen as concentration based on our previous study [26]. The optimum conditions were determined based on the percentage of encapsulation efficiency (% EE) as follows:

(1) $$\begin{array}{c} \text{Encapsulation Efficiency}\left(\%\right)=\frac{\text{Total flavonoid content in microcapsules}}{\text{Total flavonoid content in extracts}}\;\times 100\%. \end{array}$$

### 2.4. Total Flavonoid Content Determination

The calorimetric method was carried out for the determination of total flavonoid content in samples, based on the previous studies [27, 28]. Quercetin was used to create standard calibration curves for flavonoid determination. Samples (*R*. *tuberosa* L. extracts or microcapsules) were dissolved into in 5 mL of distilled water and incubated at 40°C for 45 minutes and then centrifuged at 1000 rpm for 10 min. A total of 1.2 mL of the prepared solution was mixed with 1.2 mL of 2% aluminium chloride. After mixing, the solution was incubated for 60 min at room temperature. The absorbance of the solution was measured at a wavelength of 420 nm. The total concentration of flavonoid content in the test sample was calculated from the plot of the quercetin standard curve and expressed as mg quercetin equivalent (QE)/g.

### 2.5. Alpha Amylase Inhibition Assay

All samples (extracts, microcapsules, or acarbose) prepared in various concentrations (10 to 100 *µ*g/mL for *R*. *tuberosa* L. extracts and microcapsules; 0–10 *µ*g/mL for acarbose). Next, 250 *µ*L of samples was added to 250 *µ*L of the alpha amylase enzyme solution (10 U/mL). The mixture was incubated for 10 min at 25°C. Then, 250 *µ*L of 1% soluble starch solution (w/v) was added to the mixture and incubated for 25°C for another 10 min. Finally, 500 *µ*L of DNS reagent was added, and all the mixture was incubated in boiling water for 5 min until the color of the solution changed to brownish red. The solution was cooled under running water. The solution mixture was diluted with 5 mL of distilled water and the absorbance was measured at 490 nm using a Shimadzu UV-Vis spectrophotometer. Experiments were conducted in triplicate. The percentage of inhibition activity of the alpha amylase enzyme was calculated using the following equation:

(2) $$\begin{array}{c} \text{Percentage of enzyme inhibition}=\frac{\text{Absorbance control}-\text{Absorbance sample}}{\text{Absorbance control}}\times 100\%. \end{array}$$

The IC_50_ value was calculated by drawing a linear regression equation with the sample concentration and the percentage of enzyme inhibition was plotted on the *x*- and *y*-axes, respectively. The IC_50_ value of each sample was expressed by the value of *y* = 50 and the value of *x* that was obtained as IC_50._

### 2.6. Antioxidant Activity Assay Using FRAP (Ferric-Reducing Antioxidant Power) Method

Measurement of antioxidant activity was carried out on ascorbic acid, *R*. *tuberosa* L. extract, and microcapsules. The measurement of antioxidant activity was carried out according to the method reported by [29]. The assay was initiated by adding 0.5 mL of 0.2 M phosphate buffer pH 6.6 to the samples with various concentrations, 1 to 5 *µ*g/mL for ascorbic acid, 10–50 *µ*g/mL for extracts, and 100–500 *µ*g/mL for microcapsules. Then, 0.5 mL of 1% (w/v) potassium hexacyanoferrate solution was added to the solution, and the mixture was incubated at 50°C for 20 min. After incubation, 0.5 mL of 10% (w/v) TCA solution was added. The solution was then put into a 1.5 mL test tube; 0.5 mL distilled water and 0.5 mL of 0.1% (w/v) FeCl_3_ solution were added. The solution mixture was incubated at room temperature for 5–10 min; then the absorbance was measured at a wavelength of 700 nm. Antioxidant activity is measured by the following formula:

(3) $$\begin{array}{c} \text{Percentage of antioxidant}\left(\text{\%}\right)=\frac{\text{Absorbance sample}-\text{Absorbance control}}{\text{Absorbance sample}}\times 100\text{\%}. \end{array}$$

The IC_50_ value was calculated by similar method as in alpha amylase inhibition assay, with the percentage of antioxidant activity plotted on the *x*-axis and sample concentration on the *y*-axis.

### 2.7. In Vitro Release Study

In vitro release of extracts in microcapsules was conducted in the release medium at pH of 2.2 and 7.4. The microcapsules were put in a water bath at 37°C under constant stirring at 100 rpm. Then, 10 mL of solution was taken with a time span of 30, 60, 90, and 120 min. The concentration of the compounds released from the microcapsules was calculated as a total flavonoid content as described in Section 2.5 and was expressed as percentage of the release with the following formula:

(4) $$\begin{array}{c} \text{Percentage of release}\left(\%\right)=\frac{\text{Total flavonoid content released from microcapsules}}{\text{Total flavonoid content in microcapsules}}\times 100\%. \end{array}$$

### 2.8. FTIR, Particle Size Distribution, and SEM Analysis

Samples for FTIR measurement were dried and pressed into KBR pellet before being analyzed by a FTIR spectrophotometer and measured at a range of 4000–400 cm^−1^ wavenumber, using a Fourier Transform Infrared FTIR Shimadzu-Type IR Prestige-21. The size and distribution of the microcapsules particles were determined using a CILAS 1090 Particle Size Analyzer (PSA). The shape and morphology of microcapsules were observed using a Scanning Electron Microscope (SEM) TM 3000 Hitachi, using magnification from 500x to 12,000x.

### 2.9. Data Analysis

Results were expressed as mean ± standard deviation. Statistical analyses were conducted using Statistical Package for the Social Sciences (SPSS) v.24 software [30]. The one-way analysis of variance (ANOVA) followed by Tukey's HSD test was used to determine the real difference from each variation. The differences at *p* < 0.05 were considered statistically significant.

## 3. Results

Microencapsulated *R*. *tuberosa* L. extracts were produced by spray drying. The main objective was to evaluate the manufacturer conditions that leads to the production of microcapsules with high encapsulation efficiency that still have biological activities.  Table 1 shows the encapsulation efficiency of microcapsules prepared in different pH, chitosan concentration, and stirring time. The microcapsules of *R*. *tuberosa* L. extracts prepared in pH 4, 90 min stirring time, and 0.1% (*w*/*v*) chitosan resulted in the highest encapsulation efficiency. Microcapsules prepared in these conditions were then analyzed further.

**Figure 1:**
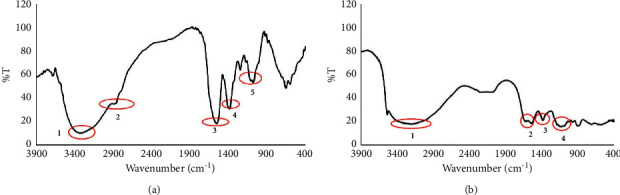
FTIR spectra of the (a) *R*. *tuberosa* L. extract. (b) Microcapsules of *R*. *tuberosa* L. extract prepared in pH 4, 90 min stirring time, and 0.1% (w/v) chitosan.

The identification of the microcapsules of *R*. *tuberosa* L. was first analyzed by the FTIR.  Figure 1 displays the FTIR results, and the assignment of the peaks of interest is tabulated in Table 2. The major characteristic peaks of the *R*. *tuberosa* L. bioactive molecules observed were 3408.75 cm^−1^ (O-H stretch), 2963.22 cm^−1^ (alkane C-H stretch), 1588.07 cm^−1^ (C=C stretch), 1414.49 cm^−1^ (C-C stretch), and 1096.25 cm^−1^ (C-O-C stretch) (Figure 1(a)). Some of these peaks were present in microcapsules. However, the FTIR spectrum of the microcapsules showing a shift in the O–H functional group at peak number 1, which initially was at wavenumber 3408.75 cm^−1^ then shifted to 3246.2 cm^−1^. Peak number 2 in Figure 1(a) was absent in Figure 1(b). Peak number 4 in Figure 1(b), at 1207.44 cm^−1^, a new absorption peak, suggests the presence of an N–H group from amine. This little peak is suggested from chitosan, which was used as coating materials for microcapsules. The absorption peak at 1118.71 cm^−1^ shows a phosphate group (P=O), suggesting this peak was from NaTPP, as a crosslinker in the preparation of microcapsules.

**Figure 2:**
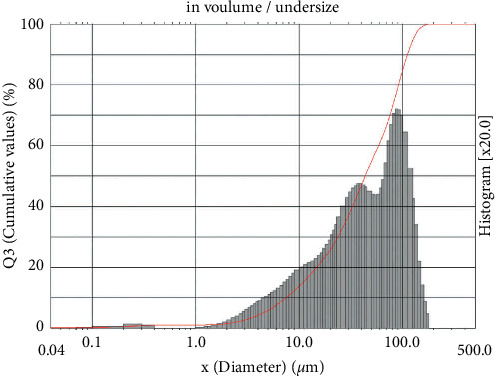
Particle size distribution from microcapsules of *R*. *tuberosa* (L.) extracts prepared in pH 4, 0.1% (w/v) chitosan concentration and 90 min stirring time. The mean diameter was 53.41 *μ*m.

**Figure 3:**
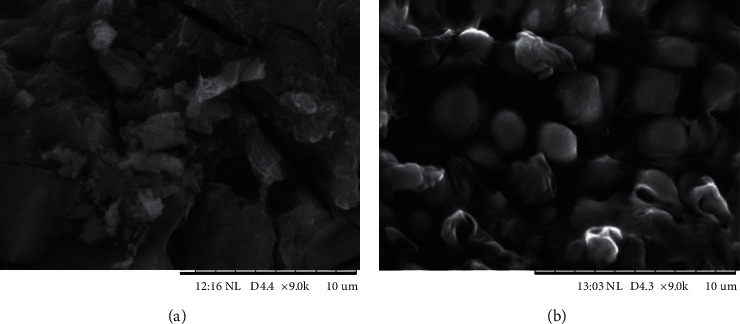
SEM images of the (a) *R*. *tuberosa* (L.) *extracts*; microcapsules of *R*. *tuberosa* (L.) extracts prepared in pH 4, 0.1% (w/v) chitosan and 90 min stirring time. The magnification was 9000x.

The obtained microcapsules were then characterized by their sizes and surface structures. Particles' size distribution analysis can be helpful to understand the microcapsules behavior and to presume its stability. The mean average size of the particles was 53.41 *μ*m, as shown in Figure 2. The SEM image is shown in Figure 3. Regarding SEM images (Figure 3), the microcapsules present more spherical and regular shapes, when compared to the morphological shapes of the *R*. *tuberosa* L. extract (Figure 3). However, the surface of the microcapsules was partially rough. Nevertheless, the viscosity of the microcapsules was not determined; this is limitation of the study.

**Figure 4:**
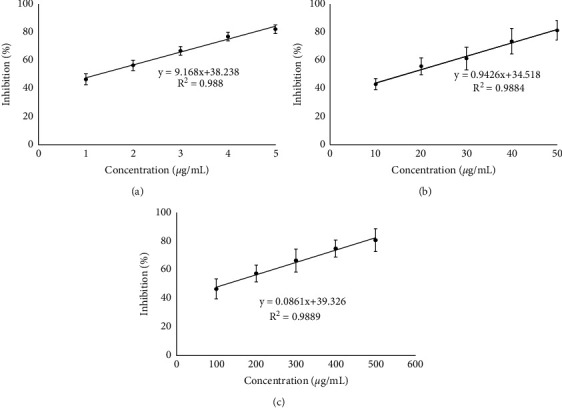
The antioxidant activity of (a) ascorbic acid, with IC_50_ value of 1.28 ± 0.23 *μ*g/mL; (b) *R*. *tuberosa* (L.) extract, with IC_50_ value of 16.42 ± 2.12 *μ*g/mL; and (c) microcapsules of *R*. *tuberosa* (L.) extract prepared in pH 4, 0.1% (w/v) chitosan concentration and 90 min stirring time, with IC_50_ value of 123.97 ± 9.33 *μ*g/mL.

**Figure 5:**
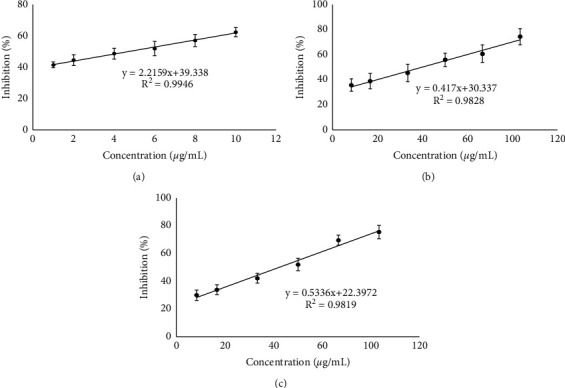
The alpha amylase inhibition of (a) acarbose, with IC_50_ value of 4.81 ± 0.34 *μ*g/mL; (b) *R*. *tuberosa* (L) extract, with IC_50_ value of 47.15 ± 4.77 *μ*g/mL; and (c) microcapsules of *R*. *tuberosa* (L.) extract prepared in pH 4, 0.1% (w/v) chitosan concentration and 90 min stirring time, with IC_50_ value of 50.65 ± 9.17 *μ*g/mL.

The biological activities of the microcapsules were tested by antioxidant and alpha amylase inhibition analyses. Figures 4 and 5 are showing results from the *in vitro* biological assay. Ascorbic acid and acarbose were used for the positive control for antioxidant and alpha amylase inhibition assay, respectively, while extract of *R*. *tuberosa* L. (nonencapsulated) was also tested for the comparison with the microcapsules.

Ascorbic acid had the highest antioxidant activity among others (IC_50_ value of 1.28 ± 0.23 *μ*g/mL). This is expected, since the ascorbic acid or vitamin C is a potent antioxidant and free-radical scavenger [34]. The microcapsules resulted in lower antioxidant activity (IC_50_ value of 123.97 ± 9.33 *μ*g/mL) than antioxidant activity of the extracts (16.42 ± 2.12 *μ*g/mL).

The *R*. *tuberosa* L. extract had an IC_50_ value of 47.15 ± 4.77 *µ*g/mL against alpha amylase, whereas acarbose as a reference had a lower IC_50_ value of 4.81 ± 0.34 *μ*g/mL. The microcapsules prepared in optimum conditions resulted in the comparable alpha amylase inhibition activity with that in the extracts, with IC_50_ value of 50.65 ± 9.17 *μ*g/mL.

**Figure 6:**
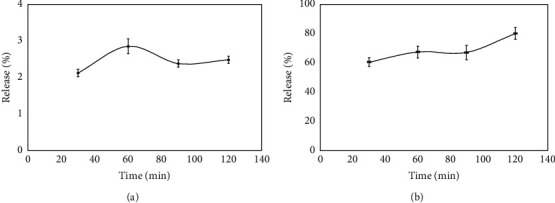
Graphs of bioactive compounds released from microcapsules of *R*. *tuberosa* (L.) extract prepared in pH 4, 90 min stirring time, and 0.1% chitosan: (a) pH 2.2 and (b) pH 7.4.

The microcapsules then underwent an in vitro released analysis.  Figure 6 shows the release profiles of microcapsules in two different mediums, pH 2.2 and pH 7.4. In Figure 6, release of extracts at pH 2.2 was smaller than the release of extracts at pH 7.4. For example, in 30 min, pH 2.2 release of the extracts was 2.12%, and in 120 min was 2.48%. At pH 7.4, microcapsules released 60.40% and 79.90% of their active compounds in 30 and 120 min, respectively.

## 4. Discussion

The crude extract of *R*. *tuberosa* L. containing multiple different groups of bioactive compounds. These compounds have bioactivities and physiological functions, such as antioxidant, antidiabetic, and antimicrobial activities [6, 25, 35].

In the previous studies [8, 36], the point of focus has been on elucidating the mechanism of action and the phytochemicals of *R*. *tuberosa* L. extract for *in vivo* diabetes treatment. Microencapsulation is increasingly in demand in controlled release because it is relatively at ease in the delivery system [11]. The use of this technique, among others, is to control the release of active compounds from medicinal ingredients; thus, the active compounds are protected from their environment or from unwanted effects, such as the influence of light, humidity, and oxygen [11, 37, 38].

In this study, spray-drying process was used to produce microcapsules from *R*. *tuberosa* L. extract. This process is the most commonly drying method used to prepare microcapsules based on chitosan. In the microcapsules' formulation, various aspects, including pH, concentration of the core material, or stirring time, play a role in the microcapsules' properties [11, 37, 39]. The optimal conditions were chosen based on the encapsulation efficiency.

Microcapsules prepared in pH 4 resulted in the highest encapsulation efficiency. The protonation process in acid environment to neutral solution can take place due to the pKa value of amino group in chitosan (∼6.5) [14]. An increase in solubility of chitosan can be made despite the protonation of amino group in acidic solution. The practice of this property has a great importance in biomedical applications when the chitosan is used to deliver drugs to acidic environment targets [15]. In pH 4, chitosan received more proton donors; as a result, more amine groups (NH_2_) will be protonated to NH_3_^+^  ions, which will ionically cross-link with P_3_O_10_^5−^ ions from NaTPP, forming microcapsules. The large number of protonated chitosan will further increase chitosan ability to absorb the bioactive compounds from the extracts, and as a result, this will increase the encapsulation efficiency.

This observation was very similar to a previous report [40] that demonstrated pH 4 was the most effective pH for designing chitosan-containing microcapsules. At pH 4, biopolymers' charge densities of opposite sign were stoichiometrically balanced. If the pH was too low, the ionization degree of amine groups on chitosan molecular chain did not facilitate the formation of homogeneous particle in the system. When pH values were higher than 4, both ionization degree and solubility of chitosan decreased, which probably induced the heterogeneity in size distribution [41].

The optimum microcapsule condition was obtained at a chitosan concentration of 0.1% (w/v). The greater the amount of chitosan is, the more ammonium ions from chitosan can bind to the extract compounds. Further increases in chitosan concentration (0.2 and 0.3%, w/v), however, did not show improvement of encapsulation efficiency. This is because the higher the concentration of chitosan, the smaller the space between the pores, hence preventing the active ingredients penetrate to microcapsules. Similar results were shown in a previous research [42]; chitosan concentration that was optimal in limonene essential oil microencapsulated in chitosan was not the highest concentration used.

The longer the stirring time, the more homogeneous and the coating process on the microcapsules increase. This is because the longer the stirring process may break down the particles into smaller pieces and have a longer dispersion of particles aggregates [43]. In this study, microcapsules with a stirring time of 90 min resulted in the highest encapsulation efficiency. This result was in agreement with an earlier study that increasing stirring time had better distribution and better structures of microcapsules [11].

Results from FTIR analysis suggest that chitosan microcapsules were successfully loaded with bioactive molecules from the extracts by ionic crosslinking through the microprecipitation method. The presence of peaks characteristic of bioactive molecules (1556.55 cm^−1^ for C=C aromatics) on the microcapsules provides direct evidence that the bioactive molecules were successfully crosslinked to the chitosan microcapsules. Furthermore, the microcapsules characteristic peaks of 1207.44 cm^−1^ for N-H amines and 1118.71 cm^−1^ for P=O were shown, but these peaks were absent from the *R*. *tuberosa* L. extracts.

In this work, the sizes and the surfaces of the microcapsules that contained *R*. *tuberosa* L. extracts appear to be more or less similar to those of the common microcapsules. Similar results were obtained by other authors that displayed small and spherical-shape microcapsules with some irregularities and roughness, from the spray drying process [10, 16, 20]. Nonetheless, the microcapsules product was already in the range of microcapsules sizes, with the mean diameter of 53.41 *μ*m.

From the *in vitro* biological assays, the microcapsules of *R*. *tuberosa* L. extracts still had high biological activities. These were shown from the antioxidant activity and the alpha amylase inhibition assays. Measurement of antioxidant activity in this study is carried out using the FRAP method. The FRAP method is used to measure the ability of an antioxidant to reduce or donate electrons to a potassium ferricyanide complex. In this method, antioxidants will act as reducing agents that will reduce Fe^3+^ ions from the potassium ferricyanide complex [K_3_Fe(CN)_6_] to Fe^2+^ [29].

The antioxidant activity of all samples is in the order of ascorbic acid > *R*. *tuberosa* L. extract > microcapsules of *R*. *tuberosa* L. extract. The potency of samples as alpha amylase inhibitor is in the order of acarbose > *R*. *tuberosa* L. extract > microcapsules of *R*. *tuberosa* L. extract.

Ascorbic acid has the strongest antioxidant activity among others. Ascorbic acid as a reducing agent can act as a donor of single reducing equivalents (H or H^+^ + e^−^) cycling between the fully reduced ascorbic acid and the radical anion, monodehydroascorbate [33]. In addition, acarbose is a known oral antihyperglycemic drug acting as alpha amylase competitive inhibitor [44].

From these results, the biological activities of the microcapsules of *R*. *tuberosa* L. are lower than nonmicroencapsulated extract. These may be caused that the bioactive compounds contained in the microcapsules cannot be released thoroughly and some compounds retained in the microcapsules. Furthermore, the microencapsulation main purpose is not to increase the biological activity, but to protect and to control the release of the active compounds. Nevertheless, microcapsules of the *R*. *tuberosa* L. extracts still active to be used as antioxidant and as inhibitor for alpha amylase. This means that microcapsules of the *R*. *tuberosa* L. extract has potential as a natural antidiabetic agent.

The microcapsules' quality is closely related with their ability to retain the core material until they reach the target site, which can be evaluated by performing release test. In the current work, microcapsules of *R*. *tuberosa* L. extracts were placed in pH 2.2 and 7.4 mediums, and the amounts of extracts released were analyzed in different time variations.

The release behaviors of extracts from the microcapsules have reflected a remarkable dependence on pH value at 37°C. In pH = 2.2, the amount of extract released is described as an initial burst (about 2%); thereafter, almost no further extract is dispersed out from the microcapsules. The results showed that the microcapsules exhibited lower diffusion coefficient for extract in an acidic medium. The initial burst release behavior can be described as the fast diffusion of extract compounds in the surface layer of the microcapsules. At pH below the pKa of chitosan (∼6.5), most of the carboxyl groups were in the form of COOH, thus reducing the electrostatic repulsion, and the intermolecular hydrogen bond would restrict the relaxation of polymer chains [15, 45].

On the other hand, the amount of released extract in pH = 7.4 reached 79.93% within 120 min. This higher release rate may be related to the weak hydrogen bonding interaction between extract and polymer network in neutral phosphate buffer; the carboxyl groups became ionized (COO^–^groups), which would induce electrostatic repulsion among negative charges in the chitosan microcapsules [14, 45]. This would cause the chitosan-NaTPP network to expand and increase the free volume spaces in the matrix, thus a higher diffusion coefficient was observed. Similar results have also been reported previously, showing that bioactive compounds was easily more released in the neutral pH than in acidic pH [11, 45, 46].

## 5. Conclusion

Microencapsulation of *R*. *tuberosa* L. extracts was successfully studied using spray drying technique with chitosan and NaTPP as wall materials. The optimum conditions were found to be at pH 4, 0.1% (*w*/*v*) chitosan, and 90 min stirring time. The FTIR analysis showed that cross-linking was developed between chitosan and NaTPP. The morphological analysis by SEM indicated that the surface of encapsulated extracts was rough and mostly spherical; nevertheless, PSA showed that the micro-sized particles were achieved at mean diameter of 53.41 *μ*m. The microcapsules of *R*. *tuberosa* L. extract still maintained their biological activities as antioxidant and as inhibitor for alpha amylase, with the IC_50_ values of 50.65 *μ*g/mL and 123.97 *μ*g/mL, respectively. The in vitro release analysis shows that *R*. *tuberosa* L. extracts were more easily released at pH 7.4 rather than when they were released at pH 2.2. These indicate that *R*. *tuberosa* L. extract was strongly bound to chitosan-NaTPP at low pH when is uncharged and hence it is expected to favor hydrophobic over hydrophilic microcapsules. This work supports the important function of microencapsulation in manufacturing plant extracts that still have high biological activities.

## Figures and Tables

**Table 1:** Encapsulation efficiency of microcapsules of *R*. *tuberosa* L. prepared in various pH, stirring time, and chitosan concentration.

Sample^*∗*^	% EE^*∗∗*^	Sample^*∗*^	% EE^*∗∗*^	Sample^*∗*^	% EE^*∗∗*^
Microcapsules prepared in pH 3	19.61 ± 0.30^b^	Microcapsules prepared in 60 min stirring time	9.21 ± 1.20^a^	Microcapsules prepared in chitosan 0.05% (w/v)	12.84 ± 2.19^c^

Microcapsules prepared in pH 4	22.51 ± 1.23^c^	Microcapsules prepared in 90 min stirring time	31.8 ± 5.34^c^	Microcapsules prepared in chitosan 0.10% (w/v)	51.81 ± 8.15^d^

Microcapsules prepared in pH 5	7.21 ± 0.76^a^	Microcapsules prepared in 120 min stirring time	9.95 ± 1.21^b^	Microcapsules prepared in chitosan 0.20% (w/v)	7.10 ± 0.91^b^

Microcapsules prepared in pH 6	6.24 ± 0.89^a^	Microcapsules prepared in 150 min stirring time	8.89 ± 1.33^a^	Microcapsules prepared in chitosan 0.3% (w/v)	6.89 ±0.87^a^

^*∗*^Samples in variation of pH prepared in 1% chitosan concentration and 60 min stirring time; samples in variation of stirring time prepared in pH 4 and 1% chitosan concentration; and samples in variation of chitosan concentration prepared in pH 4 and 90 min stirring time. ^*∗∗*^Different notations indicate significant differences between condition using the one-way ANOVA test with a level of confidence *α* = 5%.

**Table 2:** Assignment of the FTIR spectra.

Peak number	Assignment for *R*. *tuberosa* L. extract [31, 32]	Assignment for microcapsules of *R*. *tuberosa* L. extract [31–33]
1	3408.75 cm^−1^ for O-H alcohol	3246.2 cm^−1^ for O-H alcohol
2	2963.22 cm^−1^ for C-H aliphatic	1556.55 cm^−1^ for C=C aromatics
3	1588.07 cm^−1^ for C=C aromatics	1415.75 cm^−1^ for C-H alkanes
4	1414.49 cm^−1^ for C-C aromatics	1207.44 cm^−1^ for N-H amines; 1118.71 cm^−1^ for P=O
5	1096.25 cm^−1^ for C-O-C ether	

## Data Availability

The data used to support this study are included in the article and will be made available upon request.
